# The Relationship Between Staffing, Nurses' Emotional Exhaustion, and Adverse Patient Events in Critical Care Units in Sultanate of Oman

**DOI:** 10.1155/jonm/1977327

**Published:** 2025-02-18

**Authors:** Sulaiman Al Sabei, Mohammed Qutishat, Leodoro Labrague, Omar Al-Rwajfah, Ikram Burney, Raeda AbulRub

**Affiliations:** ^1^Fundamentals and Administration Department, College of Nursing, Sultan Qaboos University, P.O. Box 50, Al-Khodh, Muscat 123, Oman; ^2^Community and Mental Health Department, College of Nursing, Sultan Qaboos University, P.O. Box 66, Al-Khodh, Muscat 123, Oman; ^3^School of Nursing and Healthcare Leadership, University of Washington-Tacoma, Washington, DC, USA; ^4^Adult Health Nursing Department, Al Al-Bayt University, Mafraq, Jordan; ^5^Medical Oncology, Sultan Qaboos Comprehensive Cancer Care and Research Center, Muscat, Oman; ^6^Community and Mental Health Nursing Department, Faculty of Nursing, Jordan University of Science and Technology, Irbid 22110, Jordan

**Keywords:** adverse events, critical care units, emotional exhaustion, staffing

## Abstract

**Background:** Ensuring safe practices remains a top priority for healthcare policymakers. However, limited evidence has examined the link between individual, work-related factors, and patient safety within critical care units in Oman.

**Aim:** To assess the relationship between staffing levels, job-related emotional exhaustion, and adverse patient events among nurses working in critical care units.

**Design:** A cross-sectional study was conducted to collect data from critical care nurses in Oman. Nurses were recruited using a stratified proportional sampling method.

**Results:** A total of 694 critical care nurses participated in the study. More than half (64.1%) of the critical care nurses experienced higher levels of emotional exhaustion. Significant predictors of adverse patient events included nurse staffing level (*r* = 0.09, *p* < 0.001), emotional exhaustion (*β* = 0.25, *p* < 0.001), hospital type (being affiliated with nonteaching hospitals) (*p*=0.021), and nationality (*β* = −0.15, *p* < 0.001).

**Conclusion:** The occurrence of nurse-reported adverse events was associated with several key variables, including nurse staffing levels, emotional exhaustion, hospital type, and nationality.

**Implications for Nursing Management:** To improve patient safety, healthcare policymakers should prioritize optimizing nurse staffing levels and implement strategies to reduce emotional exhaustion, particularly in nonteaching hospitals and among specific nurse demographics.

## 1. Introduction

Ensuring patient safety is a top agenda for healthcare policymakers. Patient safety has received much attention in Middle Eastern countries owing to the large number of preventable incidences that were considered a burden for all healthcare systems and consequently necessitated surveillance [1]. Nurses play a vital role in ensuring safe practices. In Oman, the nursing profession has developed significantly, with approximately 20,000 nurses working in healthcare facilities, including 15,217 in the Ministry of Health (MOH) (2020).

In Oman, the healthcare sector has been focusing on scientific learning and research in the health field, with the establishment of the Nursing Department in 1979 and the Health Science Institute in 1982. The Specialized Nursing Institute was established in 2001, offering nursing specializations, such as midwifery, adult critical care, emergency, and infection control [1]. In 2016, the nursing diploma program offered by Oman Health Science College was upgraded to a bachelor's degree level. Furthermore, the Department of Nursing Affairs at the MOH was upgraded to the General Directorate of Nursing [1]. Since then, the General Directorate has been working on advancing the profession of nursing at different levels and aspects, such as professional and ethical aspects, developing policies, regulations, strategies, and plans to meet healthcare service requirements.

Due to this rapid growth in healthcare and the nursing profession, the WHO and numerous other international organizations have recognized Oman's remarkable growth in the past 40 years, particularly in healthcare [2]. These advancements have reshaped Oman's demographic and epidemiological landscape, leading to increased life expectancy and an increase in physical and mental health issues [3].

This has put significant pressure on healthcare providers, especially nurses, to cope with various stressors and unpleasant outcomes due to factors such as witnessing patient suffering, exposure to end-of-life care, emotional exhaustion (EE), heavy workloads, limited resources, career development expectations, and interpersonal collaboration [1, 4]. Hence, improving the nursing work environment is critical for promoting high-quality patient care.

Staffing management is an essential organizational issue that affects the working environment. Staffing involves the planning and deployment of trained human resources to meet the needs of the patient population [5]. Research has demonstrated that adequate staffing is linked to improved work satisfaction and fewer unfavorable patient outcomes such as medication errors, pressure ulcers, patient falls, fractures, and death [6, 7].

Other critical factors that can affect safe patient care are nurses' well-being and job-related EE. EE has been defined as a sense of being emotionally drained and exhausted by one's job (Maslach et al., 1996). It develops gradually and is more common in stressful workplaces such as oncology units [8]. When an individual is experiencing EE, it results in poor motivation for and interest in work, indifference toward their work, and finding no positive feelings toward their work [9, 10].

An examination of EE prevalence among nurses working across hospitals, nursing homes, and home healthcare settings in Canada revealed 44% [11]. An extensive review of the literature demonstrated that EE affects 20%–81% of healthcare providers in Arab countries as well [12]. In Oman, a study conducted among 160 nurses showed that 71% reported high levels of EE [13].

Strong evidence has demonstrated an inverse relationship between EE and safe practices, indicating that as nurses experience high levels of EE, patient safety deteriorates [14]. However, evidence on the relationship between nurses' EE, staffing levels, and patient safety in Oman is limited. Therefore, this study aimed to assess the relationship between staffing levels, nurses' job-related EE, and adverse patient events (APE) in critical care units in Oman.

## 2. Materials and Methods

### 2.1. Study Design

A descriptive correlational cross-sectional multicenter design was used in the present study.

### 2.2. Setting and Sampling Procedure

A proportional stratified clustered sampling approach was employed to select hospitals across various regions of Oman. Hospitals were stratified according to type (public or private) and location (governorate). The sample encompasses all the geographical areas of Oman, covering 11 governorates. The sample was proportionate to the affiliation of the healthcare hospitals (MOH, non-MOH, and private hospitals). At the individual level, convenience sampling was used from the participating hospitals. Registered Omani and non-Omani nurses with at least a diploma degree in nursing and 1 year of experience were eligible to participate in the study. Nurses working in areas other than critical care units were excluded from the study.

### 2.3. Sample Size

The sample size was calculated using a G-power prior sample size calculator [15]. This method utilized the predetermined statistical power, significance level, and the total number of predictors that were included in the multivariate regression analysis [16]. This method for sample size calculation has been widely used in the literature [17, 18]. In the current study, the sample size was estimated based on including 10 predictors in the regression model, 80% statistical power, 0.05 significance level, and 0.02 effect size. The required sample was 818 participants.

### 2.4. Data Collection Procedure and Ethical Considerations

The study was approved by the Institutional Review Boards of the affiliated University (SQU-EC/067/19), the MOH, and selected private hospitals (MOH/CSR/18/10,004). The hospital and nursing administrators were briefed on the purpose and importance of the study. A research assistant recruited the participants during various shifts. Participation was voluntary, and those who agreed to participate were provided with a survey packet that included a letter outlining the study's purpose, significance, and the instruments used for the variables, all placed in a return envelope. Written informed consent was obtained from the participants before data collection commenced. The participants were instructed to place their completed surveys in a locked box within their units. Participants were assured that their responses would remain confidential and that the results would be reported in aggregates. Data were collected over three months between October 2019 and January 2020.

### 2.5. Instruments

Four instruments were used, including a demographic form, nurse staffing, Maslach Burnout Inventory, and APE scale. A demographic form was designed to collect information on the participants' age, sex, educational level, nationality, years of work experience, marital status, place of work, and position.

Nurse staffing was measured by dividing the average number of patients reported by nurses in their last shift by the average number of nurses assigned to that unit. The predictive validity of this measure is supported by previous research [19, 20].

EE was measured using the EE subscale of the Maslach Burnout Inventory (Maslach et al., 1996). EE is a 9-item, 7-point Likert scale, wherein one refers to “never” and seven refers to “every day.” Higher scores indicated higher levels of EE. EE is described as a mean composite score and categorized into three levels: low (scores less than 18), moderate (scores between 19 and 26), and high (scores of 27 or more) [21]. The EE Scale has been used internationally and validated in diverse cultures, including Oman [22]. The internal consistency reliability of the scale has been well demonstrated, with a Cronbach's alpha of 0.92 [19]. In the current study, the Cronbach's alpha was 0.73.

APE, defined as damage or complications that cause patient disability or even death and happen to patients due to medical care not related to patients' pre-existing conditions, were assessed using the APE scale [23]. This scale includes items about “complaints from patient and family complaints, verbal abuse, falls, nosocomial infections and medication errors.” Participants rated each item according to the frequency of occurrence during their shifts over the past year using a seven-point Likert scale (0 = *never* to 6 = *daily*). This scale has been reported to have adequate internal reliability and criterion validity [23, 24]. The alpha coefficient for the scale in this study was 0.85.

### 2.6. Data Analysis

Statistical Package for Social Sciences software version 22 (IBM SPSS) was used for the analysis. Participants' background variables were analyzed using descriptive statistics. Pearson's correlation coefficient was used to examine the relationships between study variables. Stepwise regression was used to examine predictors of APE.

## 3. Results

### 3.1. Sample Demographics

A total of 694 critical care nurses participated in the study, with an 84.4% response rate. The sample was predominantly female (85.4%) and worked full time (92.2%). On average, nurses spent 11.9 years in the nursing profession and 7.39 years in their current unit. More than half of the sample (50.3%) were expatriates, held a diploma in nursing degrees (50.3%), and worked in teaching hospitals (54.6%). Please see Table 1 for the detailed characteristics of the nurses.

### 3.2. Description of Staffing, EE, and APE

Nurses care for 7.10 (SD = 8.76) patients on average on each shift. More than half of the nurses (64.1%) reported high levels of EE, whereas 22.9% reported moderate EE levels. The most frequently reported APE were patient and family complaints (*M* = 1.44, SD = 1.65), followed by patient and family verbal abuse (*M* = 1.29, SD = 1.52), while patient falls were the least reported adverse event (*M* = 0.59, SD = 1.13) (Table 2).

The findings of the correlational analysis showed a significant positive correlation between nurse staffing and EE (*r* = 0.06, *p*=0.01). Similarly, there was a positive significant correlation between APE and the nurse-to-patient staffing ratio (*r* = 0.09, *p* < 0.001) and nurse-perceived EE (*r* = 0.32, *p* < 0.001) (Table 3).

### 3.3. The Relationship Between Staffing, EE, and APE

The findings from multiple regression analyses identified a few variables that contributed to APE. Among the different demographic variables, nationality was identified as a strong predictor of non-Omani nurses reporting a lower incidence of adverse events than Omani nurses (*β* = −0.15, *p* < 0.001). Additionally, nurses who held a BSN degree reported fewer APE when compared to nurses who held a diploma in a nursing degree (*β* = −0.11, *p* < 0.001). Nurses working in nonteaching hospitals reported a higher incidence of APE than those who worked in teaching hospitals (*p* < 0.02). With regard to work characteristics, the nurse-patient ratio was associated with a significant increase in nurse-reported APE (*β* = 0.03; *p*=0.02). Finally, EE was associated with an increase in nurse-reported APE (*β* = 0.25, *p* < 0.001) (Table 4).

## 4. Discussion

This study aimed to assess the relationship between staffing levels, nurses' EE, and APE. The results of this study indicate that more than 60% of critical care nurses experience high levels of EE, consistent with previous studies [24], Li et al., 2024. Critical care nurses may experience high EE levels due to the intense emotional and physical demands of their roles, including dealing with life-threatening situations, managing complex patient needs, and working long irregular shifts [25]. Although precursors of EE among critical nurses were not assessed in this study, other studies identified insufficient staffing levels, long working hours, a lack of resources and support, high patient acuity, limited opportunities for professional development, and undesirable work environments as important precursors of EE among nursing professionals in Oman [26–29]. Additionally, the demanding nature of the job, the emotional stress from dealing with patients' suffering, and a lack of organizational support can all have a negative impact on nurses' psychological status including EE [30–32]. This result highlights the importance of implementing initiatives to support nurses, improve working conditions, ensure adequate resources, and promote self-care to reduce EE and improve the overall well-being of nurses [33].

Among the different adverse events that commonly occur in acute care settings, this study identified ‘patient and family complaints' as the most reported adverse events, followed by ‘verbal abuse.' These results support previous research conducted in the Philippines, which identified complaints and abuse by patients and their families as the most frequently reported adverse events in hospital settings [24]. Patient and family complaints often stem from long waiting times and delayed appointments [34–36], whereas verbal abuse often results from stress or dissatisfaction with healthcare workers or other patients [37, 38]. A future study exploring the types and causes of patient and family complaints is recommended. Such findings can inform policymakers of strategies necessary to improve the work environment and enhance patient quality of care.

Conversely, patient falls were the least frequently reported adverse events. This reflects the efforts of the MOH to promote patient safety in healthcare settings across the country. Given the importance of these findings, it is essential to implement strategies aimed at reducing these specific adverse events, including enhancing collaboration among healthcare professionals and nurses, encouraging active patient involvement in their care, and fostering a positive attitude among the healthcare staff [39], and measuring its effectiveness.

Consistent with previous evidence [40], this study found a significant positive correlation between nurse and patient staffing ratios and EE. This relationship can be attributed to the increased workload associated with higher nurse-patient ratios, which places considerable pressure on nurses and leads to feelings of being overwhelmed, anxious, and experiencing both physical and emotional fatigue [27]. This situation is often exacerbated by a shortage of resources, forcing nurses to work extended hours and take on additional responsibilities to meet patient care demands [26]. As a result, quality of care may decline, contributing to frustration, guilt, and inadequacy, all of which are strongly linked to occupational EE [41].

Findings from multivariate regression analyses revealed that nurse staffing ratio was a significant predictor of APE, indicating that as the number of patients assigned to each nurse increased, so did the incidence of perceived adverse events. This aligns with international studies [42, 43], which demonstrated that higher workloads can hinder effective communication and coordination among healthcare personnel, leading to more errors and adverse events. This issue is particularly acute in critical care areas, where the complexity of patient care demands intensive efforts to maintain high quality [39, 44]. To mitigate the risk of APE, it is essential to ensure adequate staffing and resources, thereby supporting safe and effective care delivery.

The findings also showed that each additional point in perceived EE among nurses working in critical care units was associated with an increased incidence of APE. This result is consistent with those of previous studies [24, 45], which highlight that higher levels of EE among healthcare professionals correlate with a greater likelihood of making errors. EE impairs nurses' ability to perform effectively by diminishing their cognitive and emotional resources, leading to reduced vigilance, increased fatigue, and impaired decision making [31]. Consequently, this deterioration in performance heightens the risk of APE, as exhausted and disengaged nurses may struggle to maintain the high standards of care required in critical settings [46]. Therefore, addressing EE through targeted interventions is crucial for improving patient safety and the quality of care in critical care environments.

Nurses working in nonteaching hospitals reported higher incidence of APE when compared to nurses working in teaching hospitals (*p*=0.02), which is in line with earlier studies [47–49]. This result may be attributed to disparities in staff training and experience, accessibility of resources and advanced medical technologies, enforcement of rigorous safety protocols, and extent of medical supervision and oversight offered in diverse hospital environments [50, 51]. This study also indicated lower reporting of APE among Omani nurses compared to non-Omani nurses (*β* = −0.15, *p* < 0.001). The disparity in the occurrence of adverse events between these two groups of nurses may be attributed to a range of factors, including disparities in training and educational standards, professional backgrounds, adherence to protocols and guidelines, communication abilities, and cultural influences [52]. Non-Omani nurses often experience cultural barriers that may influence their confidence and willingness to report incidents [53]. These barriers can lead to miscommunication or hesitancy in accurately describing adverse events, as non-Omani nurses may feel uncertain about language nuances or fear potential misunderstandings.

Finally, nurses who held BSN degrees reported fewer APE than diploma nurses (*β* = −0.11, *p* < 0.001), which is congruent with previous studies [53, 54]. Nurses with BSN degrees reported fewer APE than diploma nurses, likely because of their more comprehensive education and training, which equips them with enhanced clinical judgment, critical thinking, and evidence-based practice skills [53, 54]. BSN programs often emphasize leadership, research, and complex decision-making, enabling nurses to manage patient care more effectively, recognize potential risks, and intervene earlier to prevent adverse outcomes. Additionally, BSN-prepared nurses may be more exposed to advanced patient safety protocols equipped with adequate knowledge and tools to effectively assess complex situations, make informed decisions, and implement interventions that reduce the likelihood of adverse events [55, 56].

### 4.1. Limitations

This study has a few limitations. First, the cross-sectional nature of the study design may affect the reliability of the findings, as establishing a causal link between the study variables is not possible. Second, the use of self-reported measures for adverse events introduces potential bias as nurses' perceptions may not fully capture the objective occurrences of such events. Additionally, the sample was drawn from nurses working in acute-care hospitals, limiting the generalizability of the results to other healthcare settings, such as primary-care facilities. Future large-scale studies that include nurses working in different healthcare settings including primary, tertiary, and outpatient clinics are recommended. Finally, the study did not account for potential confounding factors, such as varying hospital policies or staffing models, which may also be linked to the incidence of adverse events.

## 5. Conclusion

The findings of this study highlight the high levels of EE experienced by critical care nurses, underscoring the intense demands of their work environment. Several variables were identified as contributing factors to the reporting of APE, including nationality, educational background, and work setting. The study findings also emphasize the critical need for improved staffing levels and burnout management to enhance patient safety and care quality. Addressing these factors through targeted interventions could mitigate adverse outcomes and improve overall healthcare delivery.

## Figures and Tables

**Table 1:** Nurse characteristics (*N* = 694).

Variables	*n* (%)
Gender	
Female	593 (85.4)
Male	101 (14.6)
Nationality	
Local	276 (39.8)
Expatriate	418 (60.2)
Highest education level	
Diploma	349 (50.3)
Baccalaureate	304 (43.8)
Master	41 (5.9)
Employment status	
Full-time	1879 (92.2)
Part-time	160 (7.8)
Hospital teaching status	
Teaching	379 (54.6)
Non-teaching	315 (45.4)
Hospital type	
Public	650 (93.7)
Private	44 (6.3)

	**Mean (SD)**

Age	34.16 (6.22)
Years of experience in the nursing profession	11.90 (6.79)
Years of experience in the present unit	7.39 (5.12)

**Table 2:** Descriptive summary of staffing, emotional exhaustion, and adverse events.

Scale/subscale	Mean	SD
Nurse-patient staffing ratio	7.10	8.76
Emotional exhaustion	30.59	10.96
Low (*n*, %)	90	13.0
Moderate (*n*, %)	159	22.9
High (*n*, %)	445	64.1
Adverse patient events⁣^∗^	4.88	5.38
Patient and family complaints	1.44	1.65
Patient and family verbal abuse	1.29	1.52
Patient falls	0.59	1.13
Nosocomial infections	0.98	1.33
Medication errors	0.60	1.13

⁣^∗^Scale composite score.

**Table 3:** Correlation between study variables.

Variables	1	2	3
1. Staffing	1		
2. Emotional exhaustion	0.06⁣^∗^	1	
3. Adverse patient events	0.09⁣^∗∗^	0.32⁣^∗∗^	1

⁣^∗^*p* < 0.05.

⁣^∗∗^*p* < 0.01.

**Table 4:** The influence of staffing and emotional exhaustion on adverse events.

Variables	Adverse patient events				
	*B*	SE	*β*	*t*	*p* value
*Demographics*					
Age	−0.02	0.05	−0.02	−0.33	0.75
Women versus men	1.96	0.39	0.13	5.02	0.14
Non-Omani versus Omani	−1.55	0.38	−0.15	−4.11	< 0.001
Level of education					
BSN	−1.09	0.29	−0.11	−3.80	< 0.001
MSN	−0.98	0.88	−0.03	−1.11	0.27
Total years of experience in the nursing profession	−0.06	0.05	−0.08	−1.09	0.28
Total years of experience in the current ward	−0.05	0.04	−0.04	−1.24	0.21

*Work characteristics*					
Staffing	0.03	0.02	0.06	2.37	0.02
Emotional exhaustion	0.12	0.01	0.25	9.10	< 0.001
Private versus public hospitals	−1.70	1.08	−0.04	−1.58	0.12
Nonteaching versus teaching hospital	0.75	0.32	0.07	2.31	0.02
Constant	3.36	1.96		1.72	0.09

*Note:* The model was controlled for place of work.

## Data Availability

The data that support the findings of this study are available from the corresponding author upon reasonable request.
